# Participation in scheduled asthma follow-up contacts and adherence to treatment during 12-year follow-up in patients with adult-onset asthma

**DOI:** 10.1186/s12890-022-01850-1

**Published:** 2022-02-15

**Authors:** Jaana Takala, Iida Vähätalo, Leena E. Tuomisto, Onni Niemelä, Pinja Ilmarinen, Hannu Kankaanranta

**Affiliations:** 1 Seinäjoki Health Care Centre, Seinäjoki, Finland; 2 Department of Respiratory Medicine, Seinäjoki Central Hospital, Seinäjoki, Finland; 3 Tampere University Respiratory Research Group, Faculty of Medicine and Health Technology, Tampere University, Tampere, Finland; 4 Department of Laboratory Medicine, Seinäjoki Central Hospital, Seinäjoki, Finland; 5 Tampere University, Tampere, Finland; 6 Department of Internal Medicine, Krefting Research Center, Sahlgrenska Academy, University of Gothenburg, Gothenburg, Sweden

**Keywords:** Asthma, Adherence, Scheduled follow-up, Primary care, Secondary care, Inhaled corticosteroids, Alcohol

## Abstract

**Background:**

Poor treatment compliance is a common problem in the treatment of asthma. To our knowledge, no previous long-term follow-up studies exist on how scheduled asthma follow-up contacts occur in primary health care (PHC) versus secondary care and how these contacts relate to adherence to medication and in participation to further scheduled asthma contacts. The aim of this study was to evaluate occurrence of scheduled asthma contacts and treatment compliance in PHC versus secondary care, and to identify the factors associated with non-participation to scheduled contacts.

**Methods:**

Patients with new adult-onset asthma (n = 203) were followed for 12 years in a real-life asthma cohort of the Seinäjoki Adult Asthma Study (SAAS). The first contacts were mainly carried out in secondary care and therefore the actual follow-up time including PHC visits was 10 years.

**Results:**

A majority (71%) of the patients had ≥ 2 scheduled asthma contacts during 10-year follow-up and most of them (79%) mainly in PHC. Patients with follow-up contacts mainly in PHC had better adherence to inhaled corticosteroid (ICS) medication during the whole 12-year period compared to patients in secondary care. In the study population, 29% of the patients had only 0–1 scheduled asthma contacts during the follow-up. Heavy alcohol consumption predicted poor participation in scheduled contacts.

**Conclusions:**

Patients with mainly PHC scheduled asthma contacts were more adherent to ICS medication than patients in the secondary care. Based on our results it is necessary to pay more attention to actualization of asthma follow-up visits and systematic assessment of asthma patients including evaluation of alcohol consumption.

*Trial registration* Seinäjoki Adult Asthma Study is retrospectively registered at www.ClinicalTrials.gov with identifier number NCT02733016. Registered 11 April 2016.

**Supplementary Information:**

The online version contains supplementary material available at 10.1186/s12890-022-01850-1.

## Background

Asthma is a chronic inflammatory airway disease with different phenotypes [1]. A large proportion of asthma cases are diagnosed at adult age [2, 3]. Remission of adult-onset asthma is rare [4, 5] and poor asthma control is a common problem despite improvements in understanding, evidence-based guidelines and asthma medications [6, 7]. Uncontrolled asthma has been shown to reduce both asthma- and general health-related quality of life (HRQoL) [8, 9], increase health care costs, the risk of asthma exacerbations and mortality [10]. Many factors may lead to poor asthma control including allergy, rhinitis, gastroesophageal reflux, smoking, obesity, problems in inhalation technique and poor adherence to asthma medication [1, 11–14]. One possible reason to adverse treatment outcome is non-participation to asthma follow-up visits and it seems to be a problem in many countries [15–18]. Issues affecting the adherence to treatment and occurrence to asthma follow-up visits may include both patient-related and health care system-related factors [19, 20].

To our knowledge, there are no previous long-term real-life follow-up studies on how scheduled asthma follow-up contacts occur in primary health care (PHC) versus secondary care and how these contacts relate to adherence to medication and in participation to further scheduled asthma contacts. Thus, the main aim of this study was to assess how scheduled asthma contacts occur, and possible differences in adherence to medication and participation to further follow-up depending on whether patients have follow-up contacts mainly in PHC versus secondary care. The second aim of this study was to identify the factors associated with non-participation to asthma follow-up visits.

## Methods

### Study design, inclusion and exclusion criteria

This real-life study was a part of Seinäjoki Adult Asthma Study [SAAS (www.ClinicalTrials.gov; NCT02733016)] which is a single-center 12-year follow-up study of 257 patients with new-onset adult asthma diagnosed between October 1999 and April 2002 in Seinäjoki Central Hospital respiratory department. [21] More than 94% of the patients diagnosed with novel asthma in the study site were recruited to the study and in 2001, the study population represented > 38% of novel diagnoses of asthma made to adults in the whole geographical area. [21] Smokers, patients with allergies or with concomitant COPD or other comorbidities were not excluded. [21] After asthma diagnosis was confirmed and medication initiated the patients were managed by their personal physicians mostly in PHC according to the Finnish National Asthma Programme [22] unless asthma severity or other respiratory diseases required monitoring in specialized care. As described previously[2, 4, 15, 21], after 12 years (mean 12.2, range 10.8–13.9) a total of 203 patients completed a follow-up visit where the participants gave written informed consent to the study protocol approved by the Ethics committee of Tampere University Hospital, Tampere, Finland (R12122). In addition to the data gathered at the diagnostic and follow-up visits, all data on asthma-related health care contacts during 12-year period was collected from PHC, occupational health care, private clinics and secondary care [2, 4, 15, 21]. The SAAS-study protocol has been published previously [21].

**Fig. 1:**
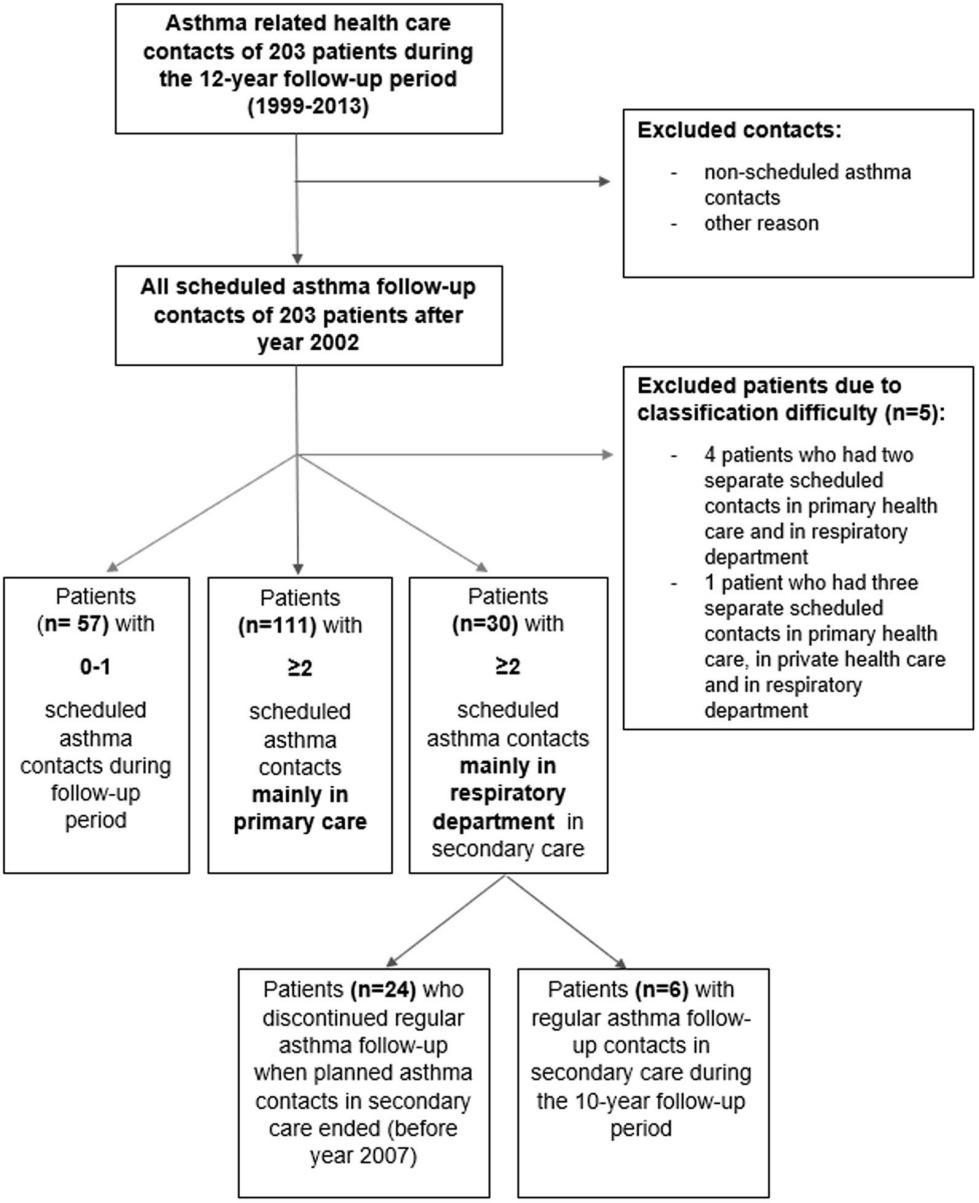
Flowchart of the distribution of scheduled asthma contacts during 10-year follow-up

In the present study all asthma-related health care contacts after asthma diagnosis of the 203 patients were explored and the data on planned asthma contacts was evaluated. Because scheduled asthma contacts during the two first years were mainly done in respiratory department, we categorized patients based on the amount of scheduled asthma contacts after 2002: 0–1 contacts vs. ≥ 2 contacts. Five patients were excluded because of classification difficulties (Fig. 1). Further analysis was performed by categorizing patients with ≥ 2 contacts into two groups according to the main location of scheduled asthma follow-up contacts (visits mainly in PHC versus mainly in secondary care) (Fig. 1). Planned follow-up contacts both in health care centres and occupational health care were considered as PHC contacts [15].

### Computation of adherence, evaluation of alcohol consumption and other clinical measurements

As described in our previous studies, adherence to inhaled corticosteroid (ICS) medication was evaluated by comparing the dispensed doses to the prescribed doses for the whole 12-year period. [23, 24] The prescribed dose in each patient was calculated based on medical records, and the dispensed ICS, short-acting β_2_-agonist (SABA) and oral corticosteroids were obtained from the Finnish Social Insurance Institution, which records all purchased medication from all Finnish pharmacies. [23, 24] The 12-year adherence and annual adherence for each patient was calculated by using specific formulas as previously described taking into account aspects from Medication possession ratio (MPR) and proportion of days covered (PDC). [23] Heavy alcohol consumption was evaluated by self-reports (according to the US definitions for alcohol consumption by portions/week), laboratory analyses [(gammaglutamyltransferase (GT) and gammaglutamyltransferase-carbohydrate-deficient transferrin-index (GT-CDT)] or by both [25, 26]. Detailed information on the assessment of asthma control and severity, lung function measurements and other clinical measurements are described separately in Additional file 1: Appendix E1.

### Statistical analysis

Continuous data is expressed as mean (SD) for variables with normal distribution. If skewed distribution, median and 25–75 percentiles are shown. The Shapiro–Wilk-test was used to assess normality. Two group comparisons were performed by using Student’s t test for continuous variables with normal distribution, Mann–Whitney test for continuous variables with skewed distribution and Pearson Chi-square test or Fisher’s exact test for categorized variables. Two-sided p-values were used. To analyse differences in annual adherence over the 12-year period between patients having scheduled contacts mainly in PHC or secondary care, annual adherence was plotted against time for individual patients and mean area under curve (AUC) values were compared by using independent-samples Mann–Whitney U test. A P value < 0.05 was regarded as statistically significant. Multivariable binary logistic regression was performed to determine the association between alcohol consumption and poor participation in planned asthma follow-up, adjusting for age, sex, pack-years, BMI and form of residency. Statistical analyses were performed using SPSS software, versions 25–26 (IBM SPSS, Armonk, NY) and GraphPad Prism software, version 9.0. (GraphPad, La Jolla, CA, USA).

## Results

### Characteristics of the study population

The included 198 patients were divided into two groups according to the number of scheduled follow-up contacts (0–1 vs. ≥ 2) during the 10-year follow-up period. After the year 2002, 141 (71.2%) patients had at least two scheduled contacts either mainly in PHC or in secondary care (respiratory department). However, 57 (28.8%) patients had only 0–1 scheduled contacts (Fig. 1).

To evaluate if differences exist in patient characteristics according to the number of scheduled contacts, we compared the patients with ≥ 2 [median 5 (interquartile range (IQR) 3–8)] scheduled contacts to those with 0–1 scheduled contacts (Table 1). At follow-up visit, mean age in both groups was 58 years. No differences were found in sex, smoking status, asthma control defined according to GINA 2010 [27] or asthma therapy steps according to GINA 2019 [28]. Patients with ≥ 2 scheduled follow-up contacts used more medication for asthma and were more often symptomatic as measured by ACT [29] and AQ20 scores. [30] The patients with ≥ 2 scheduled contacts needed more oral corticosteroid courses, collected more SABA-canisters, had a higher number of hospitalizations due to asthma and more health care visits. Patients with 0–1 scheduled asthma follow-up contacts were more often heavy users of alcohol and had higher levels of alcohol use biomarkers GT and GT-CDT (Table 1). No significant differences were found between the groups in lung function or inflammatory parameters (Additional file 1:Table E2).

**Table 1:** Characteristics of the study groups at 12-year follow-up visit

	Scheduled asthma follow-up contacts 0–1 n = 57	Scheduled asthma follow-up contacts ≥ 2 n = 141	P-value
Female n (%)	29 (50.9)	86 (61.0)	0.206
Age (y)	58 (14)	58 (14)	0.718
BMI (kg/m^2^)	27.7 (25.0–30.8)	28.1 (24.3–31.3)	0.998
Smoking history n (%)			
Ex/current	32 (56.1)	74 (52.5)	0.753
Pack-years of smokers	15 (4–31)	15 (6–28)	0.968
Chronic or allergic rhinitis n (%)	37 (64.9)	102 (72.3)	0.308
Atopic n (%) ^a^	20 (37.7)	47 (37.3)	> 0.999
Blood eosinophils (× 10^9^/l)	0.19 (0.10–0.27)	0.16 (0.10–0.28)	0.708
Uncontrolled asthma n (%) ^b^	13 (22.8)	44 (31.2)	0.477
Severe asthma (ATS/ERS 2014) n (%) ^c^	1 (1.8)	11 (7.8)	0.185
Asthma therapy steps (GINA 2019) n (%) ^d^			
Step 1–2	13 (22.8)	24 (17.0)	0.384
Step 3	11 (19.3)	33 (23.4)	
Step 4	6 (10.5)	28 (19.9)	
Step 5	9 (15.8)	30 (21.3)	
ACT score	23 (21–25)	21 (19–24)	**0.012**
AQ20 score	3 (1–6)	4 (2–7)	**0.019**
Self-reported daily ICS n (%)	34 (59.6)	117 (83.0)	**0.001**
Average prescribed daily ICS dose during 12-year follow-up (µg budesonide eq)	921 (781–1018)	1140 (944–1604)	0.308
Total adherence to ICS medication during 12-y (%)	66 (26–93)	76 (45–98)	0.259
Daily LABA in use n (%)	19 (33.3)	74 (52.5)	**0.018**
Daily SABA in use n (%)	3 (5.3)	20 (14.2)	0.089
SABA canisters (150puff/canister) during 12-y	4 (1–12)	9 (4–17)	**0.004**
Daily add-on drug in use n (%)	19 (33.3)	80 (56.7)	**0.004**
≥ 1 oral corticosteroid course for asthma during 12-year follow-up n (%)	13 (22.8)	52 (37.7)	**0.047**
Co-existing COPD (Post FEV_1_/FVC < 0.7 and pack-y ≥ 10) n (%)	13 (23.2)	20 (14.3)	0.143
Alcohol use markers above normal range n (%)			
GT	21 (37.5)	34 (24.1)	0.078
GT-CDT	15 (27.3)	20 (14.2)	**0.039**
GT-CDT	3.5 (3.2–3.9)	3.3 (3.0–3.7)	**0.021**
GT (U/I)	35.5 (28.2–67.8)	28.8 (22.1–45.5)	**0.006**
Heavy alcohol consumption (evaluated by self-report, GT-CDT index or by both) n (%) ^e^	16 (29.1)	23 (16.3)	**0.049**
≥ 1 hospitalization due to asthma n (%)	6 (10.5)	33 (23.4)	**0.048**
All asthma-related health care visits during 12-year follow-up	9 (5–16)	17 (11–24)	** < 0.001**
Scheduled asthma contacts	0 (0–1)	5 (3–8)	** < 0.001**
Unscheduled contacts ^f^	3 (0–8)	3(1–10)	0.247

Significant *p*-value shown as bold

If not otherwise mentioned shown are mean (SD) or median (25th–75th percentiles). BMI = Body Mass Index, ACT = Asthma control test, AQ20 = Airway questionnaire, ICS = inhaled corticosteroid, LABA = long-acting β_2_-agonist, SABA = short-acting β_2_-agonist, Add-on drug = long-acting β_2_-agonist, leukotriene receptor antagonist, theophylline and/or tiotropium in daily use. GT = γ-glutamyltransferase, CDT = carbohydrate-deficient transferrin, GT-CDT = combined index based on γ-glutamyltransferase (GT) and carbohydrate-deficient transferrin (CDT). ^a^ At least one positive skin prick test of common allergens. ^b^Assessment of asthma control was performed according to the Global Initiative for Asthma (GINA) 2010 report. ^c^Assessment of severe asthma was performed according to the ERS/ATS severe asthma guideline 2014. ^d^Classification of asthma therapy steps was made based on daily medication regimen according to the GINA 2019 guideline. The GINA step could not be determined in 44 patients because of the lack of medication purchased. ^e^Assessment of alcohol consumption was performed according to the US definitions for alcohol consumption by portions/week. ^f^Unscheduled contacts include visits due to respiratory infections or exacerbations

To assess whether alcohol consumption associates with poor participation (0–1 visits) in scheduled asthma follow-up contacts after adjusting for age, sex pack years, BMI and form of residency we carried out multivariable binary logistic regression analysis (Table 2). After adjustments, heavy alcohol use remained a significant risk factor for poorer participation in follow-up. Male sex showed a trend for being a risk factor for poor participation to asthma follow-up visits (Table 2).

**Table 2:** Association of various factors with poor participation in asthma follow-up (0–1 scheduled contacts) in multivariable binary logistic regression analysis

Variable	OR	95% Confidence interval	p value
Age	0.99	0.97–1.02	0.609
Male sex	1.99	0.98–4.05	0.058
BMI ≤ 24.99 (ref)	1		0.211
BMI ≥ 25–29.99	1.09	0.45–2.67	0.846
BMI ≥ 30	1.91	0.86–4.24	0.111
Pack-years ≥ 10yrs	0.79	0.74–3.52	0.228
**Alcohol heavy user**	**2.51**	**1.11**–**5.70**	**0.027**
Living alone	1.07	0.45–2.57	0.881

Significant *p*-value shown as bold

n = 192. BMI = Body Mass Index. Assessment of alcohol consumption was performed according to the US definitions for alcohol consumption by portions/week. Heavy alcohol consumption was evaluated by self-reports, GT-CDT index or by both. For men, heavy drinking is defined as consuming 14 portions or more per week. For women, heavy drinking is defined as consuming 7 portions or more per week. Portion indicates 14 g alcohol

### Comparison of patients with ≥ 2 scheduled asthma contacts mainly in PHC or in secondary care

To evaluate differences between scheduled asthma contacts carried out mainly in PHC or secondary care, the 141 patients having ≥ 2 scheduled asthma contacts were divided into groups according to the main site of asthma contacts: 111 (78.7%) patients had ≥ 2 scheduled follow-up contacts [median 4 (interquartile range (IQR) 3–7)] mainly in PHC and 30 (21.3%) patients had ≥ 2 scheduled contacts [median 4 (interquartile range (IQR) 2–5)] mainly in secondary care after year 2002 (Table 3). Scheduled contacts to private health care were rare in these groups (median 0 visits in both groups). Patients having follow-up contacts mainly in secondary care were younger, had lower FEV_1_ and FVC, higher FEV_1_ reversibility and steeper annual decline in lung function. No significant differences were found in sex, smoking status, asthma control, comorbidities, socioeconomics or health care use as shown in Table 3 and in Additional file 1: Table E3.

**Table 3:** Characteristics of the asthma patients with follow-up contacts mainly in primary health care versus secondary care

	Scheduled asthma follow-up contacts ≥ 2 mainly in PHC n = 111	Scheduled asthma follow-up contacts ≥ 2 mainly in secondary care n = 30	P-value
Female n (%)	70 (63.1)	16 (53.3)	0.400
Age (y)	60 (13)	52 (14)	**0.002**
BMI (kg/m^2^)	27.8 (23.9–31.2)	29.0 (26.3–33.5)	0.096
Smoking history n (%)			
Ex/current	57 (51.4)	17 (56.7)	0.682
Pack-years of smokers	18 (7–30)	11(4–19)	0.114
Chronic or allergic rhinitis n (%)	79 (71.2)	23 (76.7)	0.649
Atopic n (%) ^a^	34 (33.7)	13 (52.0)	0.108
Uncontrolled asthma n (%) ^b^	32 (28.8)	12 (40.0)	0.376
Severe asthma (ATS/ERS 2014) n (%)^c^	7 (6.3)	4 (13.3)	0.247
Asthma therapy steps (GINA 2019) n (%) ^d^			
Step 1–2	18 (16.2)	6 (20.0)	0.441
Step 3	30 (27.0)	(10.0)	
Step 4	23 (20.7)	5 (16.7)	
Step 5	24 (21.6)	6 (20.0)	
Co-existing COPD (Post FEV_1_/FVC < 0.7 and pack-y ≥ 10) n (%)	14 (12.7)	6 (20.0)	0.377
Number of comorbidities	1 (0–2)	1 (0–2)	0.803
Metabolic syndrome n (%)	10 (9.1)	7 (23.3)	0.054
ACT score	21 (19–24)	21 (16–23)	0.438
AQ20 score	4 (2–7)	4 (2–8)	0.783
Blood eosinophils (× 10^9^/l)	0.15 (0.09–0.26)	0.19 (0.11–0.33)	0.130
Blood neutrophils (× 10^9^/l)	3.8 (2.8–4.7)	3.5 (3.1–4.7)	0.720
Total IgE (kU/l)	57.0 (24.0–147.0)	74.5 (23.5–383.0)	0.388
FeNO (ppb)	10.0 (5.0–17.5)	10.0 (5.0–30.0)	0.863
Pre-BD FVC (%)	99.0(14.7)	91.4 (15.5)	**0.015**
Pre-BD FEV_1_ (%)	88.0 (17.5)	79.9(12.1)	**0.018**
Post-BD FVC (%)	99.9 (15.2)	93.6 (15.0)	**0.045**
Post-BD FEV_1_ (%)	91.0 (17.2)	84.4 (12.3)	0.053
Pre-BD FEV_1_/FVC	0.74 (0.68–0.79)	0.75 (0.66–0.80)	0.952
Post-BD FEV_1_/FVC	0.75 (0.70–0.81)	0.76 (0.68–0.80)	0.950
FEV_1_ reversibility (ml)	80 (10–150)	130 (55–213)	**0.013**
FEV_1_ reversibility (%)	2.89 (0.38–5.41)	4.14 (2.15 – 6.84)	0.073
Annual change in lung function from Max0–2,5 to follow-up ^e^			
FEV_1_ (ml/y)	− 39 (− 60 to − 22)	− 46 (− 76 to − 26)	0.091
FEV_1_%/y	− 0.38 (− 0.96 to 0.37)	− 0.70 (− 1.35 to − 0.15)	**0.022**

Significant *p*-value shown as bold

If not otherwise mentioned shown are mean (SD) or median (25th–75th percentiles). BMI = Body Mass Index, ACT = Asthma control test, AQ20 = Airway questionnaire, FeNO = fraction of NO in exhaled air, BD = bronchodilator, FVC = forced vital capacity, FEV_1_ = forced expiratory volume in 1 s. ^a^At least one positive skin prick test of common allergens. ^b^Assessment of asthma control was performed according to the Global Initiative for Asthma (GINA) 2010 report. ^c^ Assessment of asthma severity was performed according to the ERS/ATS severe asthma guideline 2014. ^d^Classification of asthma therapy steps was made based on daily medication regimen according to the GINA 2019 guideline. The GINA step could not be determined in 26 patients because of the lack of medication purchased. ^e^Annual change in FEV_1_ during 12 years of follow-up (ΔFEV_1_ from point of maximal lung function within 2.5 years after start of therapy to the 12-year follow-up visit)

### Changes in medication adherence over 12 years

**Fig. 2:**
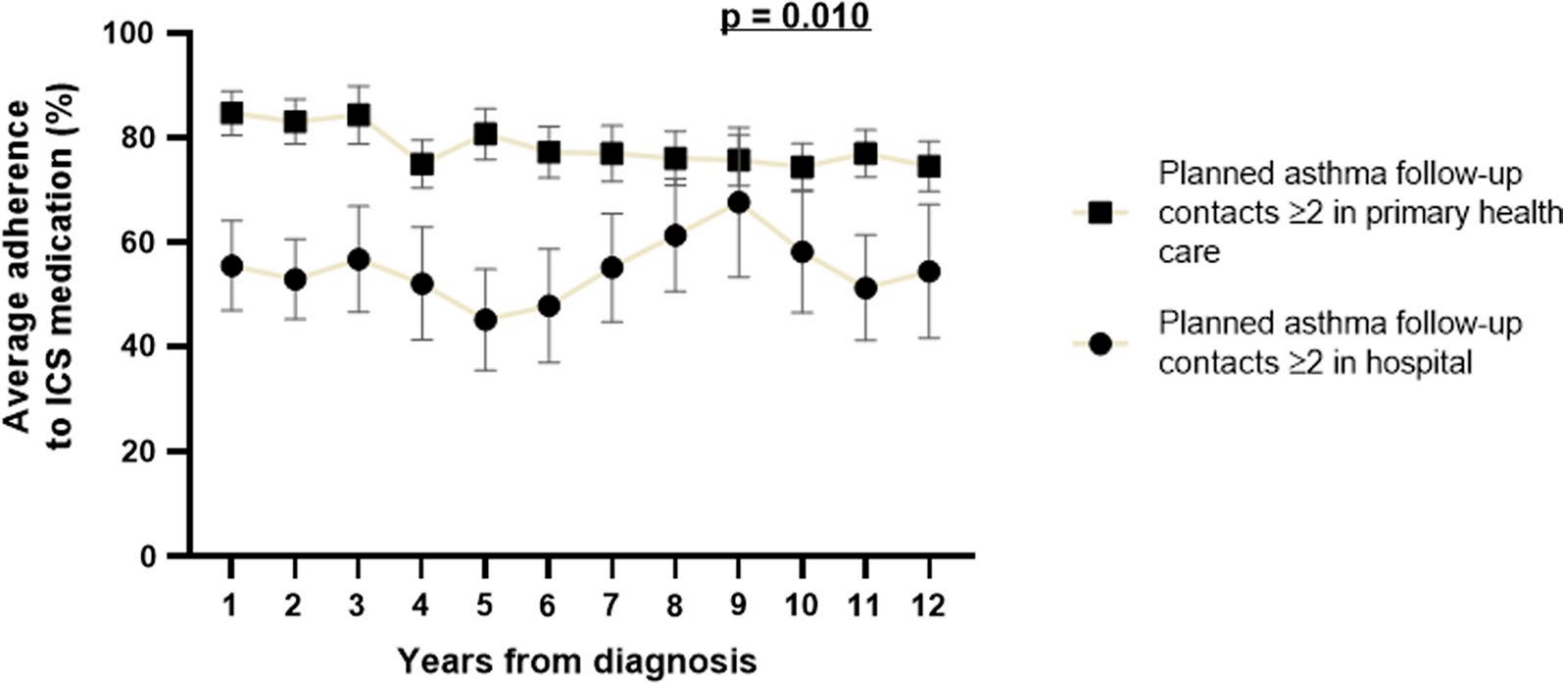
Changes in adherence over 12-years. Annual adherences shown as mean ± SEM (determined to n = 104 patients mainly in primary health care and n = 25 mainly in hospital). P-value defined by area under the curve method and independent-samples Mann–Whitney U test

Patients with ≥ 2 scheduled asthma follow-up contacts mainly in secondary care reported less often daily ICS in use and their total adherence to ICS medication was lower during the 12-year follow-up (Table 4). To explore the variation in long-term adherence, we determined annual adherence to ICS for each patient. The annual adherence was overall lower in patients with ≥ 2 scheduled asthma follow-up contacts mainly in secondary care vs. in PHC (p = 0.010) (Fig. 2). Furthermore, adherence was more stable in the group with ≥ 2 scheduled contacts mainly in PHC (annual means between 74 and 85%) and fluctuated more in the group with contacts mainly in secondary care (between 45 and 68%) (Fig. 2). In the secondary care-group the daily prescribed ICS dose (budesonide eq) was higher but no significant differences were found in average dispensed daily doses between the groups among 12-year follow-up (Table 4).

**Table 4:** Medication and adherence to ICS treatment in patients followed in primary health care or in secondary care

	Scheduled asthma follow-up contact ≥ 2 mainly in PHC n = 111	Scheduled asthma follow-up contact ≥ 2 mainly in secondary care n = 30	P-value
Self-reported daily ICS n (%)	98 (88.3)	19 (63.3)	**0.004**
Average prescribed daily ICS dose among 12-years (µg budesonide equivalents)	800 (591–1000)	967 (825–1098)	**0.008**
Average dispensed daily ICS dose among 12-years (µg budesonide equivalents)	597 (331–838)	485 (67–870)	0.197
Total adherence to ICS medication during 12-y (%)	82 (50–99)	52 (8–80)	**0.007**
Average adherence ≥ 80% during 12-years (µg dispensed / µg prescribed × 100) n (%)	54 (51.9%)	7 (28.0%)	**0.026**
Daily LABA in use n (%)	59 (53.2)	15 (50.0)	0.838
Daily SABA in use n (%)	15 (13.5)	5 (16.7)	0.768
SABA canisters (150puff/can.) during 12-y	9 (4–17)	7 (3–15)	0.322
Daily add-on drug in use n (%)	64 (57.7)	16 (53.3)	0.683
Systemic steroid in daily use (for asthma or other indication) n (%)	1 (0.9)	2 (6.7)	0.114
≥ 1 oral corticosteroid course for asthma during 12-year follow-up n (%)	39 (35.5)	13 (46.4)	0.282
≥ 2 oral corticosteroid course for asthma/2 years n (%)	20 (18.2)	3 (10.7)	0.411
Purchased oral corticosteroids prednisolone mg/year	53.6 (0–154)	63.8 (0–271)	0.498

Significant *p*-value shown as bold

If not otherwise mentioned shown are mean (SD) or median (25th–75th percentiles). ICS = inhaled corticosteroid, LABA = long-acting β_2_-agonist, SABA = short-acting β_2_-agonist. Add-on drug = long-acting β_2_-agonist, leukotriene receptor antagonist, theophylline and/or tiotropium in daily use

To explore reasons for the poorer adherence in patients with follow-up contacts mainly in secondary care (n = 30), we analyzed their scheduled follow-up contacts in more detail and found that 6 (20%) had continuous follow-up in respiratory department in secondary care during the whole follow-up period and 24 (80%) had most of their follow-up visits before the year 2007 (Fig. 1). In the latter group, only few had separate scheduled contacts in PHC, private health care or in secondary care during 2008–2013. These patients had the weakest total adherence to ICS medication (Table 5). Patients with continuous follow-up in secondary care (n = 6) had better adherence to ICS medication, more symptoms according to ACT scores, had higher therapy step according to GINA 2019, needed more SABA and had more other respiratory-related health care visits. None of them were in the working life. No significant differences were found in alcohol consumption, co-morbidities or socioeconomics (Additional file 1: Table E4).

**Table 5:** Characteristics of the patient groups with ≥ 2 scheduled asthma contacts mainly in hospital

	Scheduled asthma follow-up contacts mainly before year 2007 n = 24	Continuous asthma follow-up contacts in secondary care during the whole period n = 6	P-value
Female n (%)	13 (54.2)	3 (50.0)	> 0.999
Age (y)	49.7 (14.7)	58.8 (11.1)	0.127
BMI (kg/m^2^)	30.4 (26.8–34.6)	28.2 (19.3–28.9)	0.073
Smoking history n (%)			
Ex/current	15 (62.5)	2 (33.3)	0.360
Pack-years of smokers	13 (11)	9 (8)	0.662
ACT score	22 (18–23)	14 (10–21)	**0.033**
AQ20 score	4 (2–8)	8 (2–12)	0.321
Uncontrolled asthma n (%) ^a^	8 (33.3)	4 (66.7)	0.318
Severe asthma (ATS/ERS 2014) n (%) ^b^	2 (8.3)	2 (33.3)	0.169
Asthma therapy steps (GINA 2019) n (%) ^c^			
Step 1–2	6 (25.0)	0	**0.005**
Step 3	2 (8.3)	(16.7)	
Step 4	5 (20.8)	0	
Step 5	1 (4.2)	5 (83.3)	
Daily ICS in use n (%)	14 (58.3)	5 (83.3)	0.372
Average daily prescribed ICS dose among 12-years (µg budesonide equivalents)	921 (781–1018)	1140 (944–1604)	0.060
Average daily dispensed ICS dose among 12-years (µg budesonide equivalents)	268 (47–702)	998 (820–1714)	**0.003**
Total adherence to ICS medication during 12-y	37 (6–66)	81 (78–132)	**0.006**
Daily LABA in use n (%)	10 (41.7)	5 (83.3)	0.169
SABA canisters (150puff/canister) during 12-y	4 (2–12)	16 (12–64)	**0.009**
Pre-BD FEV_1_ (%)	79.3 (11.55)	82.3 (14.90)	0.656
Post-BD FEV_1_ (%)	83.7 (10.75)	87.3 (18.17)	0.656
Pre-BD FEV_1_/FVC	0.75 (0.68–0.79)	0.67 (0.64–0.80)	0.347
Post-BD FEV_1_/FVC	0.76 (0.68–0.81)	0.75 (0.67–0.79)	0.494
Annual change in lung function from Max_0–2,5_ to follow-up ^d^			
FEV_1_ (ml/y)	− 46 (− 86 to − 26)	− 48 (− 62 to − 25)	0.527
FEV_1_%/y	− 0.83 (− 1.5 to − 0.19)	− 0.63 (− 0.90 to − 0.45)	0.527
In working life n (%)	13 (54.2)	0 (0)	**0.024**
All asthma-related health care visits during 12-year follow-up	14 (10–22)	36 (25–55)	**0.004**

Significant *p*-value shown as bold

If not otherwise mentioned shown are mean (SD) or median (25th–75th percentiles). BMI = Body Mass Index, ACT = Asthma control test, AQ20 = Airway questionnaire, ICS = inhaled corticosteroid, LABA = long-acting β_2_-agonist, SABA = short-acting β_2_-agonist, BD = bronchodilator, FEV_1_ = forced expiratory volume in 1 s, FVC = forced vital capacity. ^a^Assessment of asthma control was performed according to the Global Initiative for Asthma (GINA) 2010 report. ^b^Assessment of severe asthma was performed according to the ERS/ATS severe asthma guideline 2014.^c^ Classification of asthma therapy steps was made based on daily medication regimen according to the GINA 2019 guideline. The GINA step could not be determined to 10 patients because of the lack of medication purchased. ^d^Annual change in FEV_1_ during 12 years of follow-up (ΔFEV1 from point of maximal lung function within 2.5 years after start of therapy to the 12-year follow-up visit)

## Discussion

In this real-life long-term follow-up study we evaluated how scheduled asthma contacts occur, assessed differences in adherence to medication and treatment compliance in PHC versus secondary care, and identified the factors associated with non-participation to scheduled contacts. We showed that 71.2% of the patients had ≥ 2 (median 5) scheduled asthma follow-up contacts during 10-year follow-up. Patients with ≥ 2 scheduled contacts used more medication for asthma, had more severe asthma symptoms and exacerbations than patients with 0–1 scheduled contacts. Patients with 0–1 scheduled contacts (28.8%) were more often individuals with heavy alcohol consumption, which also predicted poorer participation in scheduled asthma follow-up contacts in adjusted analysis. Of those with ≥ 2 scheduled asthma contacts, 78.7% had their asthma follow-up mainly in PHC. Patients with follow-up contacts mainly in secondary care (21.3%) were younger, had poorer lung function, showed more FEV_1_ reversibility and had weaker long-term adherence to ICS medication, and most of them seemed to discontinue the regular asthma follow-up when they should have arranged follow-up contacts in PHC.

According to guidelines asthma patients should have regular reviews by health care providers [1, 22, 31]. Non-adherence to regular asthma follow-up has been a common problem worldwide [15–18]. Our previous results from SAAS 12-year follow-up confirmed that only every third asthma patient attended a planned contact with health care professional in PHC each year [15]. In this study the patients with ≥ 2 scheduled asthma contacts had median 5 (IQR 3–8) scheduled contacts during 10-year follow-up resulting in approximately one scheduled contact every second year. Our results suggest that these patients may have had more difficult-to-treat asthma since they used more medication for asthma but still had more symptoms, exacerbations, and other asthma-related health care contacts. This suggests also that some of them could have benefited from more asthma phenotype-adjusted treatment. Our results are in line with previous studies [9, 16, 32, 33], showing that frequent scheduled contacts were not associated with asthma control and that patients with more symptomatic asthma participated more regularly in follow-up and used more health care services. Patients with severe asthma symptoms should be systematically reviewed to find out if they have true severe asthma or difficult-to-treat asthma [7, 34]. Based on our results it could be hypothesized that with scheduled asthma follow-up contacts more severe asthma can be treated to the same level with milder and less-symptomatic asthma.

Younger age [10, 17] and clinical features of less severe asthma [14, 17] have been suggested as risk factors for not only non-adherence to medication but also for a tendency for such patients to be lost during follow-up [17]. Also older age, low socio-economic status, obesity and ischemic heart disease are considered to be risk factors to non-participation in asthma follow-up. [35] In this study, out of 198 patients with new-onset adult asthma, 29% had only 0–1 scheduled asthma contacts during the 10-year follow-up period after the first follow-up visits in respiratory department. Alarmingly, 29 out of 203 patients did not have any scheduled contacts. [15] Asthma remission was rare (3%) in SAAS-study population [4] suggesting that it did not explain less frequent follow-up visits. It could be argued that these patients may have over-estimated their asthma control [36]. We found that patients with 0–1 scheduled contacts were more often heavy alcohol drinkers and heavy alcohol consumption associated with poorer participation in scheduled asthma follow-up contacts in multivariable binary logistic regression analysis. Assessment of alcohol consumption is not included in current asthma guidelines [1, 31] though alcohol is known to have negative impact also on respiratory health [37], treatment adherence and self-care-behavior [38, 39].

One of the main objectives of the Finnish National Asthma Programme (1994–2004) was to strengthen the role of PHC in the prevention, diagnosis and long-term therapy of asthma. [22] Our results are in line with previous study [40] showing that after implementation of the National Asthma Programme most of the adult-asthma patients were managed in PHC. Patients (21%) with ≥ 2 scheduled asthma follow-up contacts mainly in secondary care had poorer lung function, showed more FEV_1_ reversibility and the prescribed ICS doses were higher than in patients having follow-up contacts in PHC. The annual decline in lung function was also steeper. These findings suggest that patients with mainly secondary care contacts had more difficult asthma needing respiratory specialist consultation [1, 31].

It has been suggested that the major predictors of good adherence include regular asthma reviews by health care professionals and positive beliefs about the medication. [32] Adherence to medication varies in many studies between 30 and 70%. [41] Previously, we have shown that the mean 12-year adherence to ICS medication in SAAS-cohort was 69%. [23] In this study the mean 12-year adherence was 76% if patient had ≥ 2 scheduled asthma contacts and 66% in patients having only 0–1 scheduled contacts. Patients with mainly PHC follow-up contacts had better adherence (82%) to ICS medication during the whole 12-year follow-up period than those with mainly secondary care contacts (52%). In SAAS-study population adherence to ICS decreased most rapidly during the first 4 years of follow-up. [23] In the current analysis, we showed that, surprisingly, the decrease in adherence was most prominent in those being followed in secondary care. It could be suggested that PHC is more able to promote compliance in asthma treatment than secondary care. It was found that most of the patients (80%) having scheduled contacts mainly in secondary care seemed to discontinue the regular asthma follow-up when asthma was brought to control and monitoring was transferred to PHC where patients should have arranged follow-up contacts by themselves. These patients had low total adherence to ICS medication (37%). These results suggest that a proportion of patients followed in secondary care may have suffered from a more general difficulty to adhere to therapy and follow-up and may have some challenges in life management that we were not able to identify. Not only physical but also various mental health factors can interact and affect asthma outcomes. [13] Thus, it could be suggested that some of the psychological or behavioral characteristics or comorbidities of the patients monitored mainly in secondary care may both be the original reason for choosing secondary care follow-up but also the reason for non-compliance with treatment and follow-up.

Our study has several strengths. The diagnosis of asthma was made by respiratory physician and the diagnosis was based on typical symptoms and objective lung function measurements showing reversibility of airway obstruction. [21] Smokers and patients with concomitant COPD or other comorbidities were not excluded [21]. Prevalence of rhinitis and smoking among the present population was shown to be quite similar to that in a previous population-based study (FinEsS) from the same geographical area [42, 43] while the incidence of COPD was lower in the FinEsS study (9.7%) when compared to our study (16.7%). [42] This may be explained by the exclusion of patients over 70 years of age and patients´ underreporting of COPD in the previous study based on self-reports. [42] In the present study, COPD was defined by objective criteria (≥ 10 pack years and post-BD FEV_1_/FVC < 0.7). The prevalence rates of high-risk alcohol consumption in the present study population (19.6%) were also well in line with the statistics in the general population. [44, 45] Therefore, this study population well represents a typical population with asthma [21, 42]. Adherence to ICS treatment was evaluated objectively by comparing the patient´s dispensed doses to the prescribed doses for the whole 12-year period. [23] Possible limitations considering adherence calculation has been described in our previous study. [23] Weakness of our study is that results may not represent entire Finland. [20] In this study we were not able to assess more precisely the content of the follow-up contacts and how systematically patients were evaluated or advised and how these factors affected asthma control, adherence to ICS medication and participation to further follow-up contacts. Possible weakness is that alcohol markers were counted only in the follow-up visit, thus we were not able to assess whether alcohol consumption changed over time. It could be assumed that tendency to use alcohol is somewhat constant habit. [44] The number of patients in the scheduled asthma follow-up contacts mainly in secondary care was low which has led to low statistical power in analyses, thus clinical studies with larger study cohorts are needed.

Based on our results it is essential to pay more attention to participation in asthma follow-up since almost one third of all asthma patients seem to be lost-to-follow-up and regular follow-up contacts are not actualized. Particularly when asthma patients are referred to PHC from secondary care further emphasis should be placed on possible recall-systems and guidance of the patient to take care of scheduling further asthma follow-up contacts. Patients with asthma should be systematically reviewed. Alcohol consumption should be assessed in asthma patients as part of routine clinical practice and this recommendation should also be included in asthma guidelines. Further studies are needed to evaluate how other essential factors affecting asthma control are taken into account in scheduled contacts.

## Conclusion

In this 12-year real-life follow-up study we showed that patients with mainly PHC scheduled asthma contacts were more adherent to ICS medication than patients in the secondary care. Patients with ≥ 2 scheduled follow-up contacts used more medication but still had more asthma symptoms, exacerbations and health care use. Almost one third of all patients were having only 0–1 scheduled asthma contact during the long-term follow-up-period. Heavy alcohol consumption was associated with poorer participation in scheduled contacts. Thus, in the future it is necessary to pay more attention to actualization of asthma follow-up visits as well as to systematic assessment of asthma patients also including evaluation of possible alcohol consumption.

## Supplementary Information

**Additional file 1**. Participation in scheduled asthma follow-up contacts and adherence to treatment during 12-year follow-up in patients with adult-onset asthma. An additional data file containing more study statistics and methodology.

## Data Availability

All data generated or analyzed during this study are included in this published article (and its Additional file 1). According to ethical permission and patient data-protection laws of Finland, single patient data cannot be made available. The corresponding author will answer more detailed inquiries.

## Abbreviations

PHC: Primary health care
ICS: Inhaled corticosteroid
SABA: Short-acting β_2_-agonist
