# Supplementary material for: Participation in scheduled asthma follow-up contacts and adherence to treatment during 12-year follow-up in patients with adult-onset asthma
Source: BMC Pulm Med. 2022 Feb 15;22:63. doi: 10.1186/s12890-022-01850-1 (PMC8845345; doi:10.1186/s12890-022-01850-1)
Supplement: Supplementary file 1 — Additional file 1. Participation in scheduled asthma follow-up contacts and adherence to treatment during 12-year follow-up in patients with adult-onset asthma. An additional data file containing more study statistics and methodology. [file 12890_2022_1850_MOESM1_ESM.docx]

**Supplementary information**

**Participation in scheduled asthma follow-up contacts and adherence to treatment during 12-year follow-up in patients with adult-onset asthma**

Jaana Takala, MD, GP^1,2,3*^, Iida Vähätalo, M.Sc.Pharm^2,3^, Leena E. Tuomisto, MD, PhD^2,3^, Onni Niemelä, MD, PhD^4,5^, Pinja Ilmarinen, PhD^2,3#^, Hannu Kankaanranta, MD, PhD^2,3,6#^

^1^Seinäjoki Health Care Centre, Seinäjoki, Finland

^2^Department of Respiratory Medicine, Seinäjoki Central Hospital, Seinäjoki, Finland

^3^Tampere University Respiratory Research Group, Faculty of Medicine and Health Technology, Tampere University, Tampere, Finland

^4^Department of Laboratory Medicine, Seinäjoki Central Hospital, Seinäjoki, Finland

^5^Tampere University, Tampere, Finland

^6^Department of Internal Medicine, Krefting Research Center, Sahlgrenska Academy, University of Gothenburg, Gothenburg, Sweden

^#^equal contribution

Corresponding author: Jaana Takala, MD, GP

Department of Respiratory Medicine

Seinäjoki Central Hospital

Hanneksenrinne 7

FIN-60220 Seinäjoki, FINLAND

e-mail: jaana.takala@tuni.fi

**Supplementary Table E1. Exclusion and inclusion criteria used in SAAS-study.**

| Inclusion criteria   - A diagnosis of new-onset asthma made by a respiratory specialist - Diagnosis confirmed by at least one of the following objective lung function measurements   - - FEV_1_ reversibility in spirometry of at least 15 % and 200 ml     - Diurnal variability (⩾20%) or repeated reversibility (⩾15%/60 l/min) in PEF follow-up     - A significant decrease in FEV_1_ (15%) or PEF (20%) in response to exercise or allergen     - A significant reversibility in FEV_1_ (at least 15% and 200 ml) or significant mean PEF change in response to a trial with oral or inhaled glucocorticoids - Symptoms of asthma - Age ≥ 15 years |
| --- |
| Exclusion criteria   - Physical or mental inability to provide signed informed consent - Diagnosis of asthma below the age of 15 years |

Reference: Kankaanranta H, Ilmarinen P, Kankaanranta T, et al. Seinäjoki Adult Asthma Study (SAAS): a protocol for a 12-year real-life follow-up study of new-onset asthma diagnosed at adult age and treated in primary and specialised care. *NPJ Prim. Care Respir. Med.* 2015; 25:15042.

**Appendix E1: SUPPLEMENTARY METHODS**

***Lung function measurements, determination of inflammatory parameters and other clinical measurements***

Lung function measurements were performed with a spirometer according to international recommendations.^1^ The annual FEV_1_ decline was calculated by measuring the change between the highest FEV_1_ measurement available during the first 2.5 years after the diagnosis and start of inhaled corticosteroid therapy (Max_0-2.5_) and FEV_1_ at the follow-up, and by dividing the sum with elapsed time. Fraction of exhaled nitric oxide (FeNO) was measured with a portable rapid-response chemiluminescent analyzer according to American Thoracic Society standards^2^ (flow rate 50 mL·s−1; NIOX System, Aerocrine, Solna, Sweden). Venous blood was collected, and white blood cell differential counts were determined. Total immunoglobulin (Ig)E levels were measured by using ImmunoCAP (Thermo Scientific, Uppsala, Sweden). Laboratory assays were performed in an accredited laboratory (SFS-EN ISO/IEC 17025:2005 and ISO 15189:2007) of Seinäjoki Central Hospital.

Patients completed Airways Questionnaire 20 (AQ20)^3^ and Asthma Control Test (ACT)^4^. Assessment of asthma control was performed according to the Global Initiative for Asthma (GINA) 2010 report.^5^ Classification of asthma therapy steps was assessed by daily medication regimen according to the Global Initiative for Asthma (GINA) 2019 guideline [Step 1-2: >0-400ug ICS as budesonide equivalents OR daily LTRA OR low-dose ICS-formoterol; Step 3: >400ug ICS as budesonide equivalents OR low-dose ICS+LABA OR low-dose ICS+LTRA; Step 4: >800ug ICS as budesonide equivalents OR medium dose ICS and at least one second controller (LABA, LAMA, LTRA, xanthine, chromones); Step 5: >800ug ICS as budesonide equivalents and at least one second controller (LABA, LAMA, LTRA, xanthine, chromones) OR biologics].^6^ Assessment of severe asthma was performed according to the ERS/ATS severe asthma guideline 2014. ^7^ Information on alcohol consumption was assessed by detailed structured questionnaires.

SABA usage was determined by counting all dispensed SABA canisters during 12-year follow-up together and dividing the sum by 150 puffs [SABA canisters (150puff/canister) during 12-y].

Heavy alcohol consumption was evaluated by self-report, GT-CDT index or by both. Assessment of alcohol consumption was performed according to the US definitions for alcohol consumption by portions/week (portion indicates 14g alcohol).^8^ For men, heavy drinking is defined as consuming 14 portions or more per week and for women, heavy drinking is defined as consuming 7 portions or more per week. Serum levels for carbohydrate-deficient transferrin (CDT) were measured by a turbidimetric immunoassay (TIA) after ion exchange chromatography (%CDT, Axis-Shield, Oslo, Norway) and plasma γ-glutamyltransferase (GT) concentration was measured using enzymatic colorimetric assay, as standardized against IFCC (International Federation of Clinical Chemistry and Laboratory Medicine). More detailed information on GT and CDT measurements and on calculating the GT-CDT index have been previously reported.^9^

**References**

1. Miller MR. Hankinson J, Brusasco V, Burgos F, Casaburi R, Coates A, et al. Standardisation of spirometry. *Eur Respir J. 2005;*26;319–338.
2. American Thoracic Society, European Respiratory Society. ATS/ERS recommendations for standardized procedures for the online and offline measurement of exhaled lower respiratory nitric oxide and nasal nitric oxide, 2005. *Am J Respir Crit Care Med.* 2005;171: 912–930.
3. Barley EA, Quirk FH, Jones PW. Asthma health status measurement in clinical practice: validity of a new short and simple instrument*. Respir Med.* 1998;92:1207–1214.
4. Nathan RA, Sorkness CA, Kosinski M, Schatz M, Li JT, Marcus P, Murray JJ, Pendergraft TB. Development of the asthma control test: a survey for assessing asthma control. J Allergy Clin Immunol. 2004;113:59-65. https://doi.org/10.1016/j.jaci.2003.09.008
5. Global Initiative for Asthma. Global Strategy for Asthma Management and Prevention. Bethesda National Heart, Lung and Blood Institute of Health, 1995. Updated 2010. https://ginasthma.org/archived-reports/ Accessed 31 Dec 2021.
6. Global Initiative for Asthma. Global Strategy for Asthma Management and Prevention. 2019. https://ginasthma.org/archived-reports/ Accessed 31 Dec 2021.
7. Chung KF, Wenzel SE, Brozek JL, Bush A,Castro M, Sterk PJ, et al. International ERS/ATS guidelines on definition, evaluation and treatment of severe asthma. *Eur Respir J. 2014;* 43:343-373.
8. Drinking levels defined. National Institute on Alcohol Abuse and Alcoholism. https://www.niaaa.nih.gov/alcohol-health/overview-alcohol-consumption/moderate-binge-drinking
9. Hietala J, Koivisto H, Anttila P, Niemelä O. Comparison of the combined marker GGT-CDT and the conventional laboratory markers of alcohol abuse in heavy drinkers, moderate drinkers and abstainers. *Alcohol Alcohol. 2006;* 41:528-533.

**Supplementary Table E2. Additional characteristics of the groups with 0-1 and ≥2 scheduled asthma follow-up contacts at 12-year follow-up visit.**

|  | Scheduled asthma follow-up contacts  0-1  n=57 | Scheduled asthma follow-up contacts  ≥ 2  n= 141 | P-value |
| --- | --- | --- | --- |
| Number of comorbidities  (COPD included) | 1 (0-3) | 1 (0-2) | 0.392 |
| Metabolic syndrome n (%) | 6 (10.5) | 17 (12.1) | >0.999 |
| Diabetes n (%) | 7 (12.3) | 22 (15.6) | 0.660 |
| Hypertension n (%) | 20 (35.1) | 47 (33.3) | 0.869 |
| Ischemic heart disease n (%) | 5 (8.8) | 15 (10.6) | 0.799 |
| Any psychiatric disease n (%) | 8 (14.0) | 19 (13.5) | >0.999 |
| Blood neutrophils (x10^9^/l) | 3.7 (3.1-5.1) | 3.7 (2.9-4.7) | 0.557 |
| Total IgE (kU/l) | 61.0 (23.5-128.5) | 58.0 (24.0-164.5) | 0.968 |
| FeNO (ppb) | 12.0 (6.0-19.3) | 10.0 (5.0-19.0) | 0.350 |
| Pre-BD FVC (%) | 96 (16) | 97(15) | 0.586 |
| Pre-BD FEV_1_ (%) | 83(20) | 86 (17) | 0.277 |
| Post-BD FVC (%) | 98 (14) | 99 (15) | 0.730 |
| Post-BD FEV_1_ (%) | 87 (19) | 90(17) | 0.381 |
| Pre-BD FEV_1_/FVC | 0.72 (0.64-0.79) | 0.74 (0.67-0.79) | 0.435 |
| Post-BD FEV_1_/FVC | 0.76 (0.66-0.81) | 0.75 (0.70-0.81) | 0.607 |
| FEV_1_ reversibility (ml) | 120 (50-190) | 90 (25-150) | 0.112 |
| FEV_1_ reversibility (%) | 3.84 (1.52-7.76) | 3.16 (0.92-6.21) | 0.154 |
| Annual change in lung function from Max₀₋₂‚₅ to follow-up ^a^   - FEV_1_ (ml/y) - FEV_1_ %/y | -49 (-80 to -26)  -0.62 (-1.22 to 0.09) | -40 (-62 to - 23)  -0.43 (-1.09 to 0.21) | 0.198  0.315 |
| Marital status n (%)   - divorced/widow | 13 (22.8) | 18 (12.8) | 0.087 |
| Living alone | 12 (21.1) | 25 (17.7) | 0.687 |
| In working life n (%) | 28 (49.1) | 63 (44.7) | 0.637 |
| Time of education ≥ 12 years n (%) | 17 (29.8) | 40 (28.8) | 0.865 |
| Monthly income (€) | 2300 (1550-3000) | 1830 (1285-2500) | 0.092 |

*If not otherwise mentioned shown are mean (SD) or median (25th -75th percentiles). FeNO = fraction of NO in exhaled air, BD = bronchodilator, FVC = forced vital capacity, FEV_1_ = forced expiratory volume in 1 s. ^a^Annual change in FEV1 during 12 years of follow-up (ΔFEV1 from point of maximal lung function within 2,5 years after start of therapy to the 12-year follow-up visit).*

**Supplementary Table E3. Socioeconomic characteristics and health care contacts of the asthma contact groups according to the site of contact (PHC or secondary care).**

|  | Scheduled asthma follow-up contacts ≥2  mainly in PHC  n=111 | Scheduled asthma follow-up contacts ≥2  mainly in secondary care  n=30 | P-value |
| --- | --- | --- | --- |
| In working life n (%) | 50 (45.0) | 13 (43.3) | >0.999 |
| Level of education n (%)   - primary - secondary - tertiary | 48 (43.2)  47 (42.3)  16 (14.4) | 7 (23.3)  19 (63.3)  4 (13.3) | 0.098 |
| Time of education ≥ 12 years n (%) | 30 (27.0) | 10 (35.7) | 0.361 |
| Monthly income (€) | 1915 (1375-2500) | 1750 (1140-2550) | 0.364 |
| Marital status (n%)   - divorced/widow | 15 (13. 5) | 3 (10.0) | 0.764 |
| Alcohol markers above normal range n (%)   - GT - GT-CDT | 25 (22.5)  14 (12.6) | 9 (30.0)  6 (20.0) | 0.471  0.375 |
| GT (U/I) | 29.0 (21.9-43.9) | 27.2 (23.3-60.8) | 0.734 |
| GT-CDT | 3.3 (3.0-3.7) | 3.3 (3.0-3.7) | 0.874 |
| Heavy alcohol consumption (evaluated by self-report, GT-CDT index or by both) ^a^ n (%) | 17 (15.3) | 6 (20.0) | 0.580 |
| Diabetes n (%) | 14 (12.6) | 8 (26.7) | 0.086 |
| Hypertension n (%) | 37 (33.3) | 10 (33.3) | >0.999 |
| Ischemic heart disease n (%) | 13 (11.7) | 2 (6.7) | 0.738 |
| Antidepressant in use n (%) | 7 (6.3) | 3 (10.0) | 0.443 |
| Any psychiatric disease n (%) | 15 (13.5) | 4 (13.3) | >0.999 |
| Systemic rheumatic disease n (%) | 3 (2-7) | 1 (3.3) | >0.999 |
| Thyroid disease n (%) | 9 (8.1) | 2 (6.7) | >0.999 |
| Painful condition n (%) | 11 (9.9) | 4 (13.3) | 0.525 |
| Treated dyspepsia n (%) | 9 (8.1) | 3 (10.0) | 0.719 |
| ≥1 hospitalization due to asthma n (%) | 25 (22.5) | 8 (26.7) | 0.633 |
| All health care visits during 12-year follow-up | 17 (11-24) | 19 (11-33) | 0.538 |
| Unscheduled visits ^b^ | 4 (1-10) | 2 (0-9) | 0.500 |

*If not otherwise mentioned shown are mean (SD) or median (25th -75th percentiles). GT=γ-glutamyltransferase, CDT=carbohydrate-deficient transferrin, GT-CDT =combined index based on γ-glutamyltransferase (GT) and carbohydrate-deficient transferrin (CDT). ^a^ Assessment of alcohol consumption was performed according to the US definitions for alcohol consumption by portions/week. ^b^Unscheduled contacts include visits due to respiratory infections or exacerbations.*

**Supplementary Table E4. Additional characteristics of the patient groups with ≥2 planned asthma contacts after 2002 mainly in secondary care.**

|  | Planned asthma follow-up contacts mainly before year 2007  n=24 | Continuous asthma follow-up contacts in hospital during the whole period  n=6 | P-value |
| --- | --- | --- | --- |
| Chronic or allergic rhinitis n (%) | 18 (75.0) | 5 (83.3) | >0.999 |
| Atopic n (%) ^a^ | 10 (52.6) | 3 (50.0) | >0.999 |
| Co-existing COPD (Post FEV_1_/FVC <0.7 and pack-y≥10) n (%) | 5 (20.8) | 1 (16.7) | >0.999 |
| Blood eosinophils (x10^9^/l) | 0.20 (0.11-0.41) | 0.16 (0.03-0.33) | 0.402 |
| Blood neutrophils (x10^9^/l) | 3.5 (3.1-4.6) | 4.1 (2.4-6.2) | 0.860 |
| Total IgE (kU/l) | 74.5(24.5-417.3) | 102.5 (14.3-772.3) | 0.781 |
| FeNO (ppb) | 10.0 (2.5-30.0) | 9.5 (5.0-28.3) | 0.896 |
| Diabetes n (%) | 5 (20.8) | 3 (50.0) | 0.300 |
| Hypertension n (%) | 7 (29.2) | 3 (50.0) | 0.372 |
| Ischemic heart disease n (%) | 1 (4.2) | 1 (16.7) | 0.366 |
| Metabolic syndrome n (%) | 4 (16.7) | 3 (50.0) | 0.120 |
| Antidepressant in use n (%) | 3 (12.5) | 0 (0) | >0.999 |
| Any psychiatric disease n (%) | 4 (16.7) | 0 (0) | 0.557 |
| Systemic rheumatic disease n (%) | 0 (0) | 1 (16.7) | 0.200 |
| Thyroid disease n (%) | 2 (8.3) | 0 (0) | >0.999 |
| Painful condition n (%) | 2 (8.3) | 2 (33.3) | 0.169 |
| Treated dyspepsia n (%) | 1 (4.2) | 2 (33.3) | 0.094 |
| Heavy consumption of alcohol *^b^* n (%) | 5 (20.8) | 1 (16.7) | 0.656 |
| Marital status n (%)   - widow/divorced - no relationship | 2 (8.3)  0 | 1 (16.7)  2 (33.3) | **0.021** |
| Living alone n (%) | 3 (12.5) | 3 (50.0) | 0.075 |
| Level of education n (%)   - primary - secondary - tertiary | 6 (25.0)  15 (62.5)  3 (13.2) | 1 (16.7)  4 (66.7)  1 (16.7) | 0.896 |

*If not otherwise mentioned shown are mean (SD) or median (25th -75th percentiles). FeNO = fraction of NO in exhaled air. ^a^ At least one positive skin prick test of common allergens. ^b^ Assessment of alcohol consumption was performed according to the US definitions for alcohol consumption by portions/week.*
